# Potent Anti-Cancer Properties of Phthalimide-Based Curcumin Derivatives on Prostate Tumor Cells

**DOI:** 10.3390/ijms20010028

**Published:** 2018-12-21

**Authors:** Silvia Belluti, Giulia Orteca, Valentina Semeghini, Giovanna Rigillo, Francesca Parenti, Erika Ferrari, Carol Imbriano

**Affiliations:** 1 Department of Life Sciences, University of Modena and Reggio Emilia, via Campi 213/D, 41125 Modena, Italy; silvia.belluti@unimore.it (S.B.); valentina.semeghini@unimore.it (V.S.); giovanna.rigillo@unimore.it (G.R.); 2 Department of Chemical and Geological Sciences, University of Modena and Reggio Emilia, via Campi 103, 41125 Modena, Italy; giulia.orteca@unimore.it (G.O.); francesca.parenti@unimore.it (F.P.)

**Keywords:** curcuminoids, prostate cancer, cell proliferation, anti-cancer drugs, phthalimide

## Abstract

Metastatic castration-resistant prostate cancer is commonly treated with chemotherapy, whose effect is less than satisfactory. This raised the need for novel agents for the treatment of prostate cancer. In the present study, five phthalimide-based curcumin derivatives were synthesized and completely characterized to assess improved stability, pharmacodynamics, and radical scavenging ability. To investigate the potential application in anti-cancer therapy, the anti-proliferative activity of the synthesized molecules was determined on aggressive prostate tumor cells. We demonstrated that the K3F21 derivative has increased potency compared to curcumin, in terms of GI50, anti-proliferative and anti-migrating activities. K3F21 inhibits anchorage-dependent and -independent growth of prostate cancer cells by altering the expression of key genes controlling cell proliferation, such as Cylins D1, B1 and B2, and apoptosis, among which Puma, Noxa, and Bcl-2 family members. Finally, the anti-cancer activity of K3F21 was demonstrated by the analysis of cancer-associated PI3K/AKT, ERK, and p38 signaling pathways.

## 1. Introduction

Prostate cancer (PCa) is the second most commonly diagnosed cancer and leading cause of cancer death in western males [1]. The progression from normal prostate to prostatic intraepithelial neoplasia (PIN) and localized adenocarcinoma occurs over many years. Adenocarcinoma is a slow-growing tumor and progression to advanced and metastatic cancer is a relatively late process. Androgen deprivation therapy (ADT), the gold standard for the treatment of advanced PCa, has initial response rates of about 80–90%, but nearly all men eventually develop castration-resistant PCa (CRPC), which is currently incurable [2]. CRPC is commonly treated with different approaches: taxane chemotherapy (such as docetaxel and cabazitaxel), secondary hormonal therapeutic agents (abiraterone and enzalutamide), cellular immunotherapy (sipuleucel-T), or radiotherapy (radium-223). Despite this, inevitable progression of disease frequently occurs [3,4]. A considerable number of novel agents are currently under investigation worldwide to cure PCa and metastatic CRPC [5].

Curcumin (1,7-bis [4-hydroxy-3-methoxyphenyl]-1,6-heptadiene-3,5-Dione), a polyphenol obtained from the rhizome *Curcuma longa* L., and its analogs have shown anti-cancer properties by suppressing tumor initiation and progression [6,7], through the modulation of multiple signaling pathways and the inhibition of cell proliferation, invasion, metastasis, and angiogenesis [8].

Curcumin has demonstrated chemopreventive and chemotherapeutic activity also in PCa. In vitro, it reduces the expression of androgen receptors (AR), which appears to enhance the progression of PCa to the hormone refractory state CRPC [9]. Experiments performed on LNCaP, PC3, and DU145, metastatic PCa cells from lymph node, bone, and brain, respectively, showed that curcumin impacts on cell proliferation by decreasing the expression of epidermal growth factor receptor (EGFR) and cell cycle cyclins. Moreover, curcumin anti-proliferative activity has been associated to increased expression of the cyclin dependent inhibitors (CDKs) p21, p27, and p16, both in vitro and in vivo. Curcumin targets various signaling pathways, among which the PI3K/AKT network, commonly constitutively activated in PCa (for a review see [10]). Interestingly, curcumin has been recently found to affect cancer associated fibroblast (CAF)-driven PCa invasion, promoted by prostate tumor–stromal interaction, through the inhibition of the MAOA/mTOR/HIF-1α signaling pathway [11]. These data pointed at curcumin as a protective molecule against the epithelial to mesenchymal transition (EMT), a highly complex process allowing the cells to escape from the primary tumor and disseminate at distant sites.

Despite the proven efficacious anti-proliferative properties of curcumin against cancer cells in vitro and in vivo, there is currently no approved health claim for this molecule [12]. The main controversial dark side of this polyphenol is its apparent instability in physiological environment. This limits a possible successful and controlled application in clinics and does not allow to fully understand which mechanisms are activated by the molecule and which by its metabolites. It is therefore crucial to identify stable derivatives and characterize their molecular basis of action against cancer cell proliferation and metastatization. Recently, Nelson et al. [13] pinpointed the main concerns in selecting curcumin as pharmaceutical lead compound. However, a wide slice of the scientific community does not completely agree with this lapidary verdict [14,15,16,17]. In this landscape, we devoted research efforts to develop new stable curcumin analogs based on phtalimide (K3F).

Phthalimide-based drugs firstly appeared in the late 1950s and Thalidomide, the most notable one, was prescribed to pregnant women as a sedative and anti-emetic agent. The benefits of this compound were soon darkened by the discovery of its teratogenicity that forced its withdrawal from market. Today, Thalidomide is used in the treatment of erythema nodosum leprosum, multiple myeloma, myelodysplastic syndrome, and shows promising properties in the treatment of autoimmune disorders [18]. Recently, the identification of the basis for its teratogenicity has allowed the development of new thalidomide derivatives without teratogenic activity [19]. Early clinical trials showed that thalidomide has clinical anti-tumor activity in hormone-refractory PCa [20], therefore the development of analogues and/or its administration in conjunction with other anti-cancer agents are under exploration in order to improve its efficacy and reduce toxicity.

Here, we describe the synthesis, chemical and pharmacokinetic characterization, and anti-proliferative activity of new phthalimide-based curcumin derivatives on human PCa cells.

## 2. Results

### 2.1. Synthesis and Characterization

**Figure 1:**
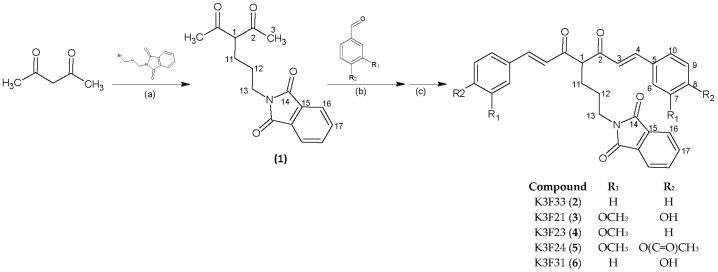
General scheme for the synthesis of phthalimide-based curcuminoids. (**a**) K_2_CO_3_/KI, dry acetone, 80 °C, 24 h; (**b**) B_2_O_3_, tributylborate, n-butylamine, DMF, 80 °C, 6 h; (**c**) 0.5 M HCl, 80 °C, 1 h.

The synthesis of curcumin-like structures is commonly performed by one-pot “Pabon reaction” [21] or its modifications [22]. The reaction proceeds through the complexation of boron by acetyl-acetone (acac), or another β-diketone, in order to protect the methylenic carbon and activate the side methyl groups as nucleophiles. In a further step, Knoevenagel condensation takes place with vanillin or other selected benzaldehydes. Finally, when the reaction is accomplished in N,N-dimethylformamide (DMF), the product separates by acidification with hydrochloric acid. In order to obtain the phthalimide-based curcuminoids (Figure 1), acac was functionalized by SN_2_ nucleophilic substitution catalyzed by K_2_CO_3_, and 2-(4-acetyl-5-oxohexyl)-1H-isoindole-1,3(2H)-dione (1) was then used as reactant in the following Pabon reaction. With respect to acac, the presence of phthalimide chain shifted the tautomeric equilibrium of compound 1 in favor of the β-diketo form, as a consequence boron complexation was slow and the protection step needed longer time (2 h vs. 30 min). The synthesized compounds, namely K3F, were isolated as yellow-orange powders by the general synthetic scheme reported in Figure 1.

pH-metric titrations were performed in order to assess K3F acidity and predict the most abundant species in physiological condition. On increasing pH, a titration trend was observed for all the compounds, and, as reported for K3F21, the plot of A vs. pH at fixed λ highlighted the presence of at least one equivalent point (Supplementary Figure S1). Overall protonation constants (β_HL_) were calculated from spectrophotometric data and optimized by means of HypSpec software [15] (Supplementary Table S1). K3F23, K3F24 and K3F33 behaved as weak monoprotic acids, with a pK_a_ value close to 8–8.50 associated to the keto-enol moiety. As for the other two ligands, namely K3F21 and K3F31, the acid-base equilibria were more complex in view of the polyprotic nature of the compounds, and three logβ_LH_ values were calculated. Species distribution curves (Supplementary Figure S2) provided reliable prediction of neutral and charged forms as function of pH, and consequently helped to envisage cell penetration and distribution in physiological conditions. For monoprotic acids K3F23, K3F24, and K3F33, the prevailing species at pH 7 was the neutral one (HL). As for K3F21 and K3F31, they behaved as less weak acids, in fact at pH 7 58% of K3F21 and 65% of K3F31 were found in the mono-dissociated negatively charged form (H_2_L^−^), these percentages increased up to 74% and 86% respectively at pH 7.4. K3F31 resulted to be more dissociated than K3F21 at the same pH, hence more acid. This outcome could be attributed to the stabilizing effect of the intramolecular hydrogen bond between the methoxyl and hydroxyl groups on the aromatic ring in the indissociated form of K3F21, which shifts the equilibrium in favor of the neutral species (H_3_L).

**Figure 2:**
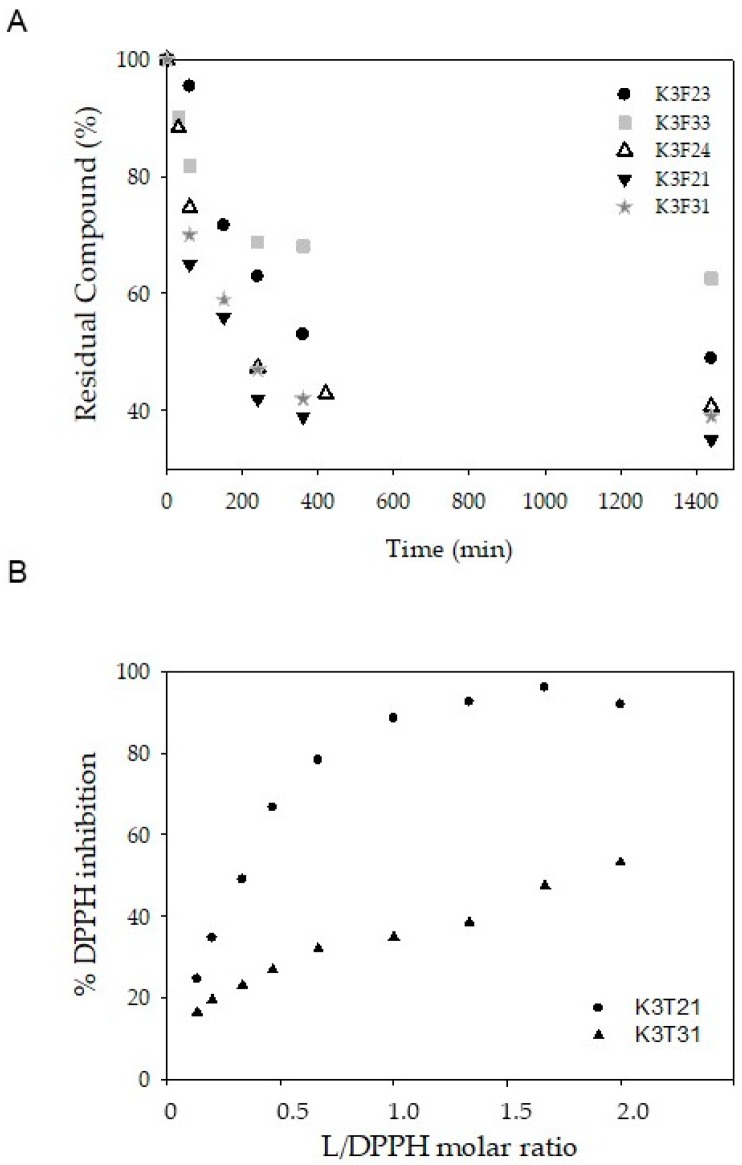
Stability and radical scavenging ability of K3F derivatives. (**A**) Residual % of K3F compounds vs. time (PBS 0.01 M, NaCl 0.1M, pH = 7.4, 37 °C in darkness). Residual % is calculated as A_t_·100/A_0_, where A_t_ and A_0_ stand for absorbance at λ_max_ at time *t* and time *zero*, respectively. (**B**) Percentage of inhibition of free DPPH radical ([DPPH^·^] = 60 µM) in the presence of K3F compounds at increasing concentrations. Compounds concentration is expressed as molar ratio (compound/DPPH).

The coupling of curcumin backbone with phtalimide moiety improved stability with respect to the lead molecule that degrades up to 40% within the first hour [23], in particular K3F33 was the most stable compound of the series, with a residual percentage close to 60% after 24 h (Figure 2A).

The phenolic derivatives K3F21 and K3F31 were tested for their radical scavenging ability by DPPH assay (Figure 2B). Both K3F21 and K3F31 exerted radical scavenging activity against DPPH radical, however K3F21 was more effective as suggested by IC_50_ values (24 µM vs. 120 µM). The intramolecular hydrogen bond between the phenolic hydrogen and the methoxyl oxygen seems to be a *conditio sine qua non* for the formation of a stable radical species.

### 2.2. Phthalimide-Based Curcumin Derivatives Decrease Viability of Human Cancer Cells

**Figure 3:**
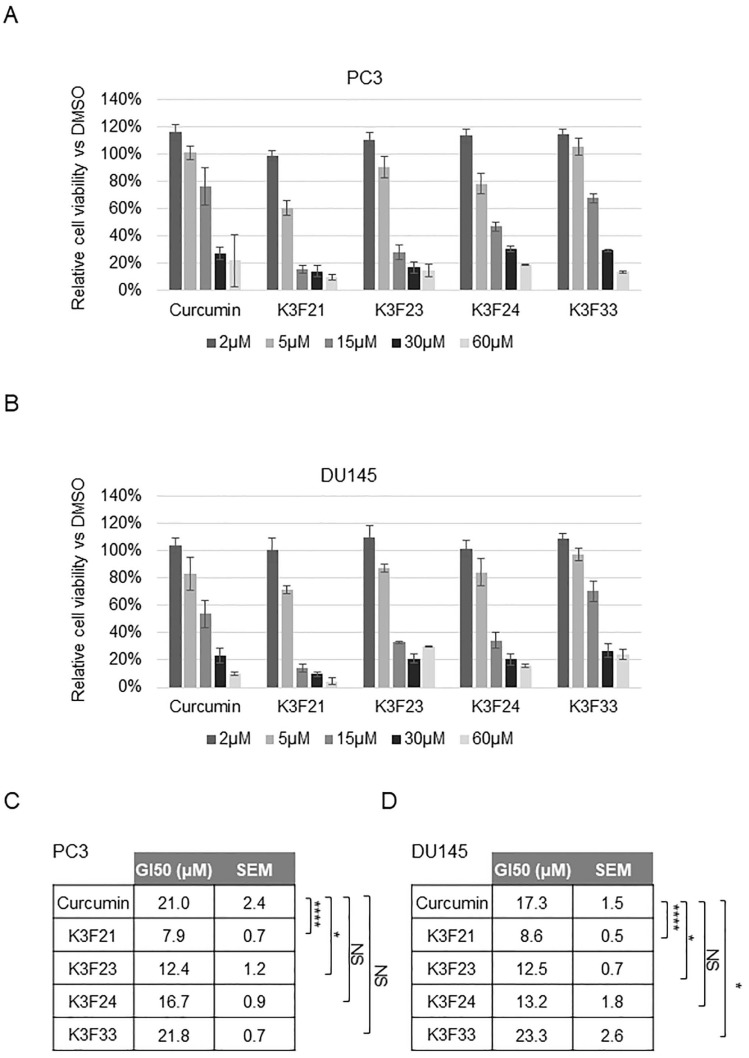
Dose-dependent effect of K3F-derivatives on PCa cells. Cell growth of PC3 (**A**) and DU145 (**B**) prostate cancer cells was assessed by MTT assays following 48 h treatment with the indicated molecules. Data are presented as relative cell viability (%) of treated cells versus DMSO control cells, arbitrarily set at 100%. The bars represent the mean of four independent experiments ± SEM. GI50 values, SEM and statistical significance compared to curcumin have been indicated for PC3 (**C**) and DU145 (**D**). *p* values * < 0.05, **** < 0.0001, NS = not significant.

To investigate the potential anti-tumor activity of the new synthesized molecules, we tested their effect on the proliferation of cancer cell lines, in particular PC3 and DU145, two of the most representative in vitro models of PCa, in which AR is not expressed. Dose–response assays were performed with K3F21, K3F23, K3F24, and K3F33 (Figure 3A,B) and GI50 values were determined as the concentration that causes 50% growth inhibition following 48 h of treatment (Figure 3C). The comparison between curcumin and K3F-derivatives highlighted that the addition of phthalimide significantly enhances the anti-proliferative activity of K3F21 and K3F23 in PC3 and DU145 cells (Figure 3C). Similarly, we tested the activity of the compounds in another cancer cell line, the colon carcinoma HCT116 cells, in which the effect of curcumin has been largely investigated and characterized [24]. Also in these cells, K3F21 is the most active molecule compared to the other derivatives (Supplementary Figure S3A,B).

**Figure 4:**
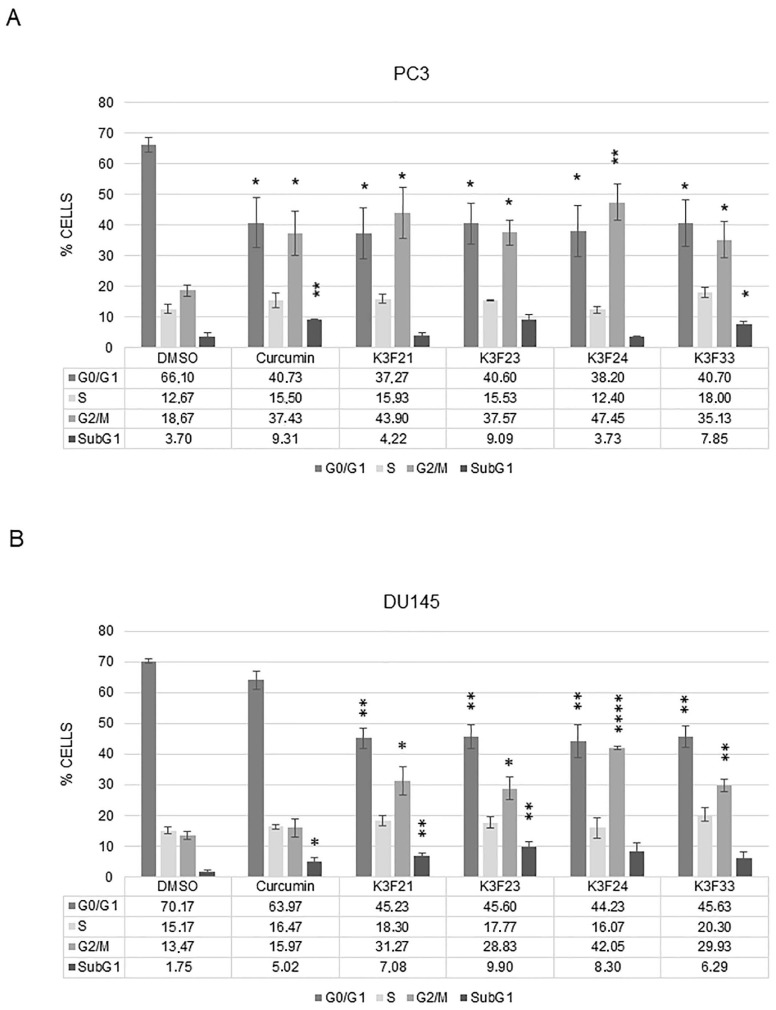
Effects on cell cycle progression of K3F derivatives. Cell distribution between cell cycle phases following administration for 48 h of DMSO, Curcumin or the indicated K3F-derivatives on PC3 (**A**) and DU145 (**B**) prostate cancer cells at GI50 concentrations. The bars represent the mean of three independent experiments ± SEM. Representative images of cell cycle analysis are shown in Supplementary Figure S4. *p* values refer to the comparison between K3F-derivatives and DMSO samples: * < 0.05, ** < 0.01, **** < 0.0001.

To determine whether K3F-derivatives could have cytotoxic rather than cytostatic activity, we analyzed the distribution of the cells within the different phases of the cell cycle following the administration of the molecules at GI50 doses for 48 h (Figure 4 and Supplementary Figure S4A,B). In PC3 cells, curcumin administration resulted in an evident increase of the G2/M population, which raised from about 19% in control cells (DMSO) to 37% in treated cells. Similarly, K3F21, K3F23, K3F24, and K3F33 doubled the percentage of G2/M cells, highlighting a cytostatic activity of the molecules. Only the administration of curcumin and K3F33 significantly increases SubG1 events, indicative of cell death (Figure 4A). As for DU145, curcumin mainly acts as a cytotoxic drug, as indicated by the significant increase of SubG1 events from 1.75% to 5% in control and treated cells, respectively. K3F24 and K3F33 showed the ability to arrest cell cycle progression, while K3F21 and K3F23 both arrested the G2/M progression and induced cell death (Figure 4B).

The semisynthetic taxane docetaxel (DTX), chemotherapeutic agent approved by FDA for its ability to prolong survival in patients with metastatic CRPC [25], was used as positive anti-proliferative compound. As expected, DTX administration at GI50 dose showed a high cytotoxic effect associated to a robust increase in SubG1 events (Supplementary Figure S4C,D).

K3F-derivatives were also tested in HCT116 cells: all the molecules induced a significant increase in SubG1 events (Supplementary Figure S3C).

Taking into consideration that all K3F-derivatives showed anti-proliferative effects, but K3F21 is the molecule with the lowest GI50 value in all the tested cell lines, we decided to further investigate its molecular activity as anti-cancer drug in PCa cells.

### 2.3. K3F21 Administration Modulates the Transcription of Genes Involved in PCa Cell Growth

**Figure 5:**
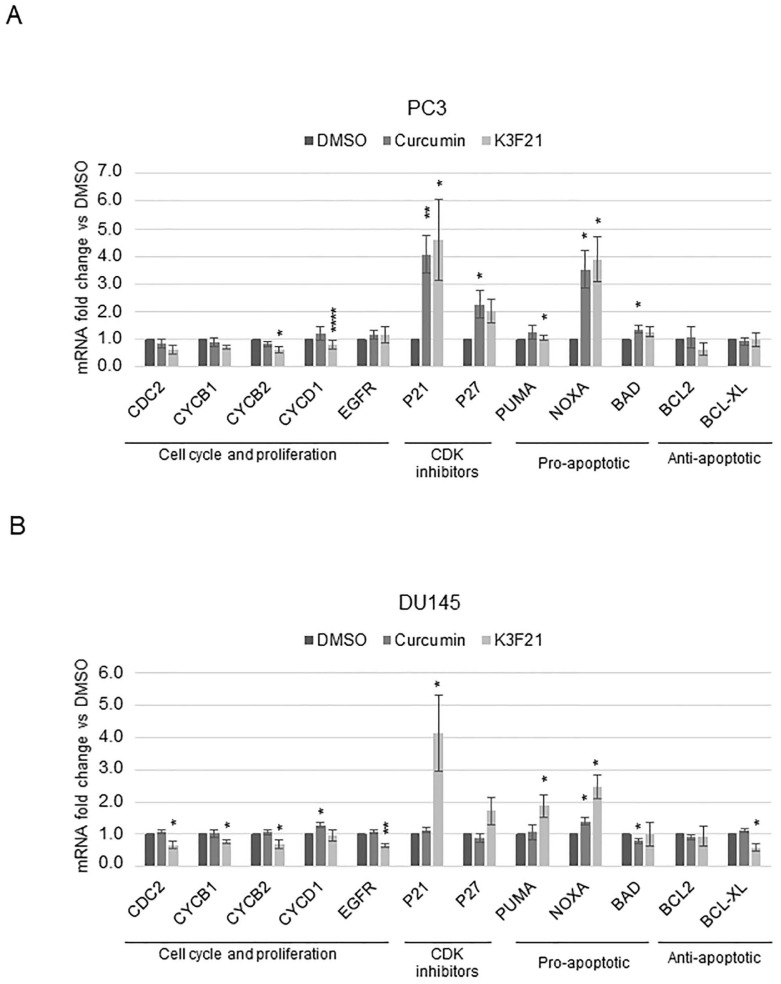
Effect of curcumin and K3F21 on gene transcription. RT-qPCR on PC3 (**A**) and DU145 (**B**) prostate cancer cells treated with DMSO, curcumin and K3F21 for 48 h at GI50 doses. Data are presented as relative transcript levels, normalized with RPL21 housekeeping gene, with DMSO levels arbitrarily set at 1. The bars represent the means of three independent experiments ± SEM. *p* values refer to the comparison between curcumin and K3F21 with DMSO samples: * < 0.05, ** < 0.01, **** < 0.0001.

The anti-proliferative and pro-apoptotic activity of curcumin on PCa cells was previously reported [10,26,27,28]. Among the mechanisms through which curcumin impacts on PCa proliferation, transcriptional modulation of genes controlling cell cycle progression and apoptosis has been described in vitro and in vivo (reviewed in [10]). We therefore decided to investigate the transcriptional effect of K3F21 administration on these processes in PC3 and DU145 cells, analyzing selected representative target genes. In both cell lines, we observed a general decrease, though not always significant, in the transcription of *Cdc2*, *CyclinB1*, *CyclinB2*, and *CyclinD1* cell cycle genes in K3F21-treated cells compared to curcumin and control cells (Figure 5). The transcription of genes encoding CDK inhibitors p21 and p27 similarly increased following curcumin and K3F21 administration in PC3 cells, while they raised only after K3F21 treatment in DU145 cells. These results are consistent with cell cycle analysis (Figure 4), showing that curcumin and K3F21 similarly arrested cell cycle progression in PC3 cells, while only K3F21 induced a G2/M block in DU145 cells. The analysis of EGFR, linked to the proliferation of PCa cells, highlighted that its expression is reduced by K3F21 only in DU145 cells. Finally, we analyzed few apoptotic genes, such as the pro-apoptotic *Noxa*, *Puma*, and *Bad*, and the anti-apoptotic *Bcl-2* and *Bcl-xl* genes. While *Noxa* was induced by K3F21 as well as curcumin, *Puma* increased exclusively following K3F21 administration, in both PCa cells. As for anti-apoptotic genes, different transcriptional programs were activated in the two cell lines: *Bcl-2*, but not *Bcl-xl*, was weakly repressed by K3F21 in PC3, as opposed to DU145.

### 2.4. K3F21 Affects the Key Molecular Pathways Promoting PCa Progression

**Figure 6:**
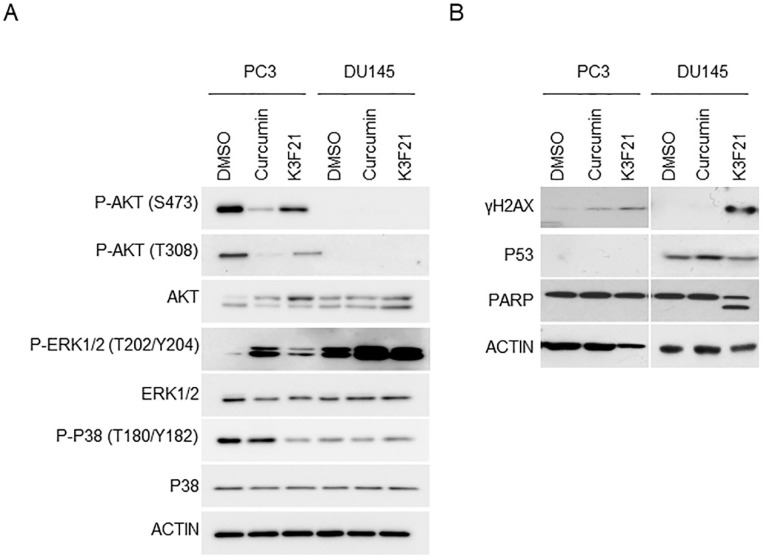
Effects of K3F21 on PCa promoting pathways. Total cellular lysates from PC3 and DU145 cells treated with DMSO, curcumin and K3F21 for 48 h at GI50 doses were analyzed by western blot with the indicated antibodies involved in receptor tyrosine kinase signaling (**A**) or DNA damage pathways (**B**). Actin was used as loading control.

Multiple intracellular signaling pathways implicated in PCa progression are modulated by anti-cancer drugs, as corroborated by the analysis of whole cellular extracts from DTX-treated PCa cells (Supplementary Figure S5). By western blot analysis, we determined whether K3F21 is able to inhibit the activation of receptor tyrosine kinase signaling (Figure 6A).

Firstly, we studied the effect of K3F21 administration on PI3K/AKT pathway activation, significantly deregulated in PCa [29]. Both curcumin and K3F21 inhibited the phosphorylation of AKT in Ser473 and Thr308 in PC3 cells [30]. We were not able to detect AKT phosphorylation in DU145 cells, presumably due to the presence of the oncosuppressor PTEN that minimizes endogenous AKT activation [30]. Administration of chemotoxic drugs has been shown to induce ERK phosphorylation, and an inverse relation seems to link ERK and AKT activity in PCa [30,31,32]. Western blot analysis showed that both curcumin and K3F21 increased ERK1/2 phosphorylation in PC3 and, more evidently, in DU145 cells, likely as a consequence of reduced basal levels of PI3K/AKT activity [30].

Finally, p38 phosphorylation was investigated as important mechanism mediating cellular pro-inflammatory responses in PCa [10]. The administration of K3F21, but not curcumin, inhibited phospho-p38 expression in PC3, while no changes were observed in DU145 cells.

We also analyzed the effect of the molecules on the expression of γH2AX, as a marker of DNA damage commonly activated by chemoterapeutic drugs (Figure 6B). While curcumin increased γH2AX in PC3 only, K3F21 triggered DNA damage in both PC3 and DU145 cells, indicating a genotoxic effect independent from the status of p53. Indeed, the oncosuppressor p53, involved in DNA damage response, is not expressed in PC3 and mutated in its activity in DU145 cells. Moreover, the evaluation of PARP-1 cleavage corroborated the activation of apoptotic cell death following K3F21 administration only in DU145 cells, as already observed by cell cycle analysis (Figure 4).

### 2.5. K3F21 Inhibits Metastatic Ability of PCa Cells to Migrate and Proliferate

**Figure 7:**
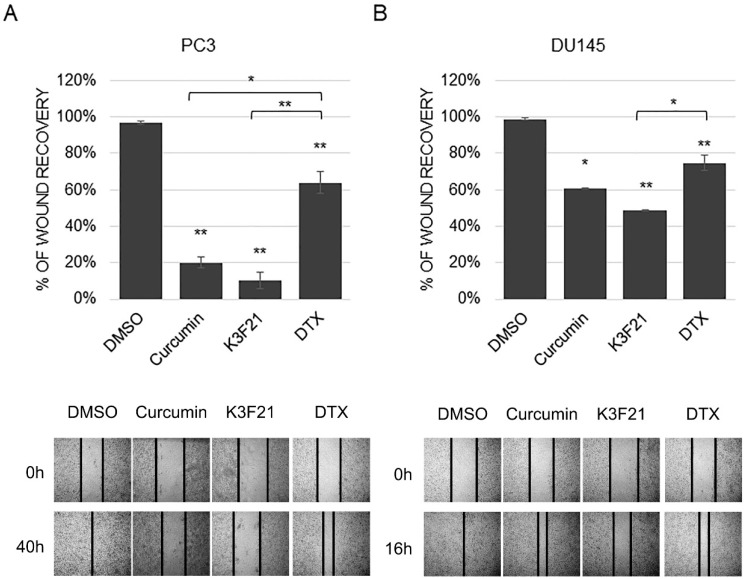
Inhibition of cell migration by curcumin, K3F21 and DTX. The percentage of wound recovery was assessed following DMSO, curcumin, K3F21 and DTX administration at GI50 doses in PC3 (**A**) and DU145 (**B**) after 40 and 16 h, respectively. Representative images are shown in lower panels. *p* values * < 0.05, ** < 0.01.

Increased cell motility of cancer cells, among which PCa cells, strongly impacts on their metastatic potential. We therefore tested whether K3F21 could decrease the migration ability of PC3 and DU145 cells, characterized by high metastatic potential. In vitro wound assays were performed and cell migration of curcumin and K3F21-treated cells was calculated as percentage of wound recovery compared to control cells. As shown in Figure 7, while DMSO-treated cells filled about 100% of the wound area, K3F21 exerted inhibitory effects on the migratory capacity of PCa cells, similarly to curcumin. Interestingly, K3F21 showed more potent anti-migration activity compared to DTX, commonly used in anti-cancer clinical therapy.

**Figure 8:**
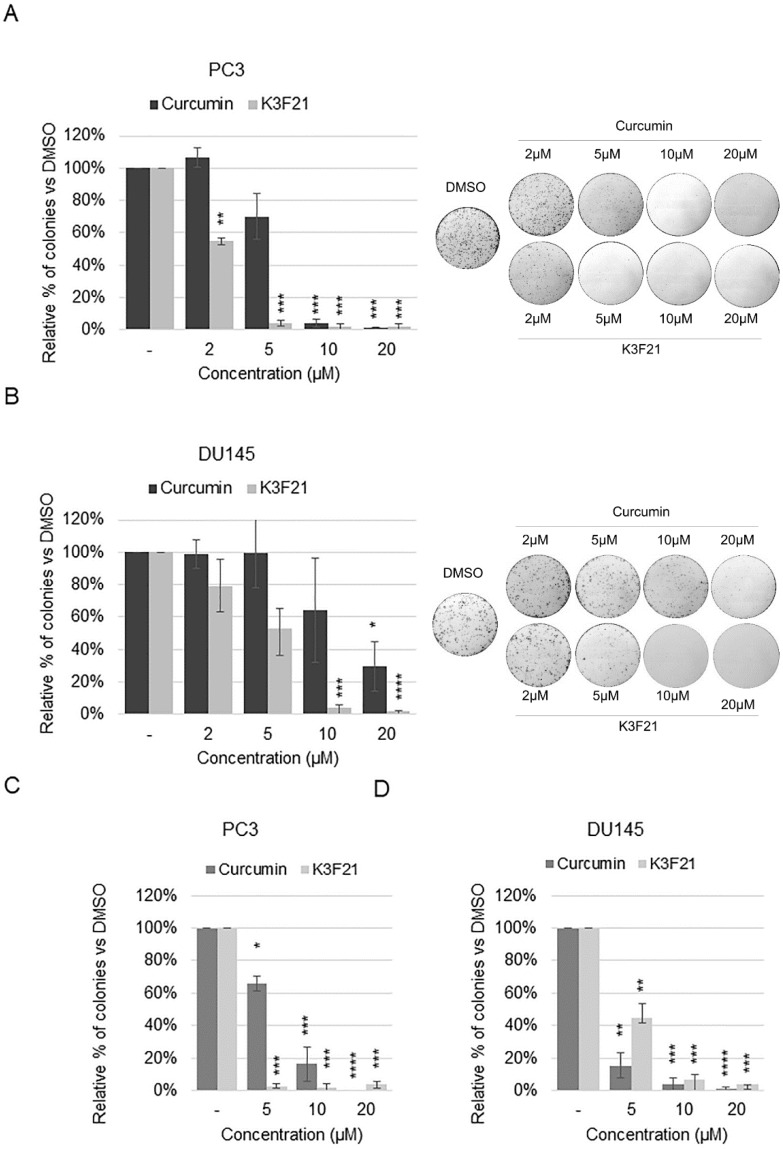
K3F21 inhibits colony formation of PCa cells. The number of colonies in PC3 and DU145 cells cultured in anchorage-dependent (**A**,**B**) or anchorage-independent conditions (**C**,**D**) in the presence of increasing doses of curcumin and K3F21 is reported as percentage versus DMSO treated cells, arbitrarily set at 100%. Data are the mean of three independent experiments ± SEM. P values refer to the comparison between curcumin and K3F21 with DMSO samples: * < 0.05, ** < 0.01, *** < 0.001, **** < 0.0001. Representative images of anchorage-independent colony assay are shown in Supplementary Figure S6.

Next, we performed a clonogenic assay, based on the ability of a single cell to grow into a colony, to determine the effectiveness of the anti-proliferative activity of K3F21 to inhibit cell growth of PCa cells also in this condition. Plated PC3 and DU145 cells were treated with different doses of curcumin and K3F21 and the number of colonies were counted following 7 or 10 days, respectively, and reported as percentage vs. DMSO control cells. K3F21 clearly inhibited cell growth when administered at lower concentrations than curcumin, in both cell lines (Figure 8A,B). Interestingly, despite the GI50 value of K3F21 in traditional 2D monolayer PC3 culture was established as about 10 µM, colony formation was quite completely abolished already at 5 µM, at least in PC3 cells.

Finally, we investigated whether K3F21 was able to inhibit the proliferation of PCa cells when cultured in suspension within a semi-solid gel. The ability to grow in the absence of anchorage to the extracellular matrix (anchorage-independent condition) is one of the hallmarks of malignancy and is crucial in the tumor progression. The decrease in colony formation reached up to 95% following K3F21 treatment at 5 and 10 µM in PC3 and DU145 cells, respectively (Figure 8C,D, Supplementary Figure S6). In PC3 cells, we observed enhanced anti-tumor activity of K3F21 compared to curcumin: similar effects were induced by curcumin when administered at 10 and 20 µM compared to K3F21 5 µM.

### 2.6. K3F21 Enhances Anti-Tumor Activity of Docetaxel in DU145 Cells

**Figure 9:**
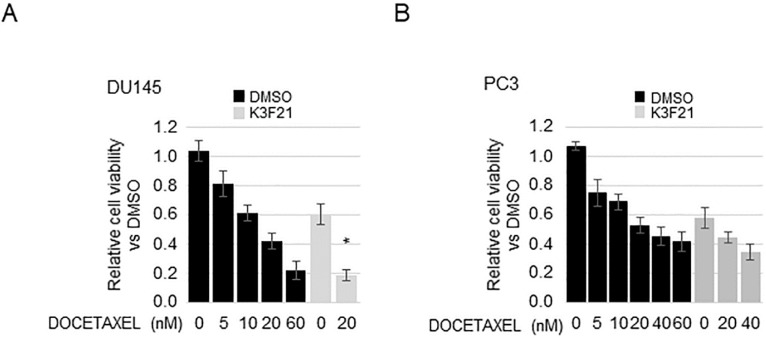
Combinatorial effect of K3F21 with docetaxel on PCa cells viability. The effect of docetaxel or docetaxel/K3F21 co-administration determined by MTT assay in DU145 (**A**) and PC3 (**B**) is indicated relatively to DMSO treatment, arbitrarily set at 1. * indicates synergism (CI < 1).

The combination of DTX with other anti-proliferative molecules has been proposed as a promising strategy for enhancing the sensitivity to chemotherapy in CRPC. Curcumin co-administration to DTX was shown to increase the anti-proliferative activity of the two molecules with respect to single treatments. In particular, their combination modulates the expression and activity of proteins commonly hyper-activated in PCa [33]. We therefore decided to investigate the effect of K3F21 co-administration with DTX on PC3 and DU145 cells (Figure 9). Treatment with DTX resulted in a dose-dependent decrease in cell viability of PCa cells. In DU145 cells, co-administration of K3F21 and DTX 20nM for 48 h induced a significant decrease in cell viability compared to independent treatments with DTX or K3F21. The determination of the Combination Index (CI) [34] revealed a slight synergism (CI = 0.8) between the drugs with a DTX drug reduction index (DRI) of 2.9, hinting that concurrent administration with K3F21 could allow an effective reduction of DTX dose. Differently, none of the evaluated K3F21/DTX combinations affected cell growth when compared simultaneously to both DTX and K3F21 single administration in PC3 cells.

## 3. Discussion

In preclinical models, thalidomide and its derivatives have shown growth inhibition and enhanced susceptibility to apoptosis of tumor cells [35]. However, in terms of the achieved response rates, i.e., the percentage of patients whose cancer shrinks or disappears after treatment, thalidomide showed mild activity towards CRPC. Phase II clinical trial showed that about 30–40% of patients with metastatic CRPC had a decline in PSA of ≥40%, associated with an improvement of clinical symptoms, following thalidomide treatment [36,37].

As regards curcumin, several studies have shown its potential therapeutic effects on PCa cells survival, both in vitro and in vivo [16,26,27,38]. In particular, the activity of curcumin has been well described on PC3 cells, accepted in vitro model of highly aggressive prostate cancer [16,39]. Nevertheless, the growth suppressive activity and bioavailability of curcumin in humans showed limitations for its use as an effective therapeutic agent in cancer. Therefore, the conjugation of phtalimide with curcumin and its derivatives could represent a successful strategy to develop new multi-target derivatives to defeat PCa. In addition, as previously observed for other β-diketo substituted curcuminoids [22], the presence of a bulky substituent in α position to the keto-enol moiety shifts the equilibrium towards the diketo form, improving stability and potentially bioavailability. K3F33, K3F23, and K3F24 show an acid-base behavior similar to curcumin, with a typical pK_a_ value ranging from 8 to 8.5 that can be attributed to the dissociation of enol proton. Differently, as suggested for other curcuminoids [40], the low value of the first pK_a_ for K3F21 and K3F31 could be reasonably attributed to the phenol group, which acidity is decreased by the conjugated keto-enol group in para position. For these compounds, the second pK_a_ is due to the keto-enol moiety, and the last one to the second phenolic group.

This study reports on the synthesis, chemical and biological characterization of the phthalimide-based curcuminoids as new potential therapeutic molecules in PCa. Chemical characterization and in vitro data on tumor cell lines identified K3F21 as the most attracting molecule for further cellular and molecular characterization of its anti-proliferative activity. In fact, K3F21 is a weak acid that is indissociated in physiological condition, hence present in its neutral form. The high lipophilicity (data not shown) together with its low molecular mass allow to predict high bioavailability. The presence of phtalimide moiety stabilizes the diketo tautomer, improving on one hand pharmacokinetics with respect to curcumin and maintaining similar radical scavenging activity on the other. K3F21 shows higher efficacy in terms of growth inhibitory activity (GI50) compared to curcumin, not only in PCa cells but also in human colon adenocarcinoma cells. Although K3F21 has a similar GI50 concentration on PC3 and DU145 cells, it exerts different anti-proliferative effects in the two cell lines. While its administration to PC3 significantly increases the G2/M population, without inducing cell death, DU145 treated cells shows both G2/M cell cycle arrest and apoptosis (Figure 4). The analysis by western blot of cleaved-PARP1 suggests the activation of a caspase-mediated cell death (Figure 6).

PC3 and DU145 cells are characterized by non-luminal-like phenotypes and are representative of aggressive disease, with similarity in overall gene expression [41]. Indeed, the analysis of single gene transcription, performed by RT-qPCR, highlighted the activation of quite similar transcriptional programs by curcumin and K3F21 in the two cell lines (Figure 5). Despite this, we should consider that PC3 and DU145 cells differ in the status of the *p53* gene, which encodes for a transcription factor involved in the response to cellular stresses, DNA damage, and cytotoxic drugs. During PCa progression, epithelial cells acquire different mutations in key genes controlling cell proliferation and cell survival, among which p53 [42]. In particular, mutations that give rise to mutant gain of function (GOF) or dominant negative (DN) p53 allow the tumor to survive anticancer therapies [43]. The administration of DNA damaging agents to mutant p53 cells may potentially further increase cancer progression: mutant p53 cells usually escape cell cycle arrest following DNA damage, which eventually triggers the acquisition of more mutations allowing cancer cell survival. While PC3 cells are p53-null, therefore they do not express p53 at the protein level, DU145 cells express mutant DN p53 (P223L/V274F) [44,45]. K3F21 efficiently inhibits cell proliferation in DU145 as well as in PC3 cells when cultured in normal 2D conditions. Differently, curcumin and K3F21 activities on cell migration and colony formation in anchorage-independent condition, key futures of cancer cells, highlighted the increased sensitivity of PC3 cells vs. DU145 cells towards curcumin and K3F21 (Figure 8). It is interesting to note that in 3D culturing conditions, that better mimic the physiological in vivo tumor growth condition, K3F21 is more active than in normal 2D monolayer culture conditions.

Finally, we should also consider the status of the PTEN/PI3K/AKT axis in the examined cells. PTEN is one of the anti-apoptotic genes frequently mutated in PCa: while PC3 are PTEN-negative, DU145 cells possess the functional protein. This difference plays a great role in the activation of the PI3K/AKT and ERK pathways: PTEN expression in DU145 cells minimizes aberrant activation of PI3K/AKT, which is sufficient for upregulation of ERK phosphorylation. ERK activation seems to be necessary for drug-induced death and its inactivation has been identified as a hallmark of advanced PCa [31,32,46]. Consistent with these published data, our western blot analysis highlighted that K3F21-induced inhibition of AKT phosphorylation is associated with increased ERK activation in both PC3 and DU145 cells (Figure 6).

Treatment of PCa cells with DTX inhibits their proliferation and activates apoptosis. Since curcumin has been recently shown to increase the efficacy of DTX treatment in PCa [33], we decided to investigate the effect of K3F21 and DTX combination. In DU145 only, we were able to quantitatively determine synergism with CI<1 between the two molecules. Moreover, the favorable DTX dose-reduction index (DRI) suggested the promising potentiality of K3F21 as a therapeutic adjunct with DTX in the treatment of PCa. Further studies will help to elucidate why this effect is observed only in DU145 and not in PC3 cells.

Overall, our results indicate that K3F21 exhibits similar or more potent activity than curcumin in the inhibition of prostate cancer cell growth and migration, as well as in the modulation of key molecular pathways involved in tumor progression and survival. These results open the opportunity to develop K3F21 molecule as a new therapeutic drug in aggressive PCa tumors.

## 4. Materials and Methods

### 4.1. General Procedures and Chemicals

Elemental analysis was performed on Thermo ScientificTM FLASH 2000 organic elemental analyzer. The percentage of C, H, and N were experimentally calculated. Liquid chromatography/mass spectrometry (LC/MS) was performed on Agilent 6300 Ion Trap LC/MS System equipped with an electrospray ionization (ESI) interface. The compounds were separated using Agilent Zorbax SB C18 30 × 2.1 mm, 3.5 µm. Samples were prepared in MeOH and diluted to 10 ppm in MilliQ water; blank was MilliQ water. Eluent phase: pump A H_2_O (formic acid 1%), pump B ACN (formic acid 1%), gradient: 10% of B for 1 min, 10–100% of B for 5 min, then 100% of B for 4 minutes, flux 0.3 mL/min, injection volume 10 µL. The ion spectra were obtained in positive mode, using a scan range between 100 and 1500 m/z. High-purity nitrogen was used as nebulizer and drying gas (flow rate 10 L/min, 350 °C). The nebulizer gas pressure was 32 psi and the capillary voltage was 3.5 kV. All reagent grade chemicals were purchased from Sigma-Aldrich (St. Louis, MO, USA) and used without further purification unless otherwise specified. NMR spectra were recorded on a Bruker FT-NMR AVANCE III HD 600 MHz spectrometer with 5 mm CryoProbe BBO H&F at 298 K. Nominal frequencies were 600.13 MHz for ^1^H and 150.9 MHz for ^13^C. For each sample, ~5 mg were weighed and diluted up to 0.6 mL with the proper deuterated solvent into 5 mm NMR tube. 90° pulse was calibrated for each sample and standard NMR parameters were used to achieve quantitative results (relaxation delay 10 s). Proton and carbon chemical shifts are given in parts per million (ppm) vs. external TMS, and were determined by reference to the solvent residual signals. Typical 2D homo- and hetero-nuclear techniques were used for assignment, i.e., ^1^H,^1^H-COSY, ^1^H,^13^C-HSQC, ^1^H,^13^C-HMBC. The purity of all final compounds was determined to be at least 95% pure by a combination of LC-MS, NMR, and combustion analysis.

### 4.2. Synthesis

2-(4-acetyl-5-oxohexyl)-1H-isoindole-1,3(2H)-dione (1), (2-((4Z,6E)-5-hydroxy-7-(4-hydroxy-3-methoxyphenyl)-4-((E)-3-(4-hydroxy-3-ethoxyphenyl) acryloyl) hepta-4,6-dien-1-yl)isoindoline-1,3-dione (K3F21) and 2-((4Z,6E)-4-cinnamoyl-5-hydroxy-7-phenylhepta-4,6-dien-1-yl)isoindoline-1,3-dione (K3F33) were synthesized and characterized as previously reported by the authors [47]. The same procedure was applied for the synthesis of K3F23, K3F31 and K3F24 that are here completely characterized.

(2-((4Z,6E)-5-hydroxy-7-(3-methoxyphenyl)-4-((E)-3-(4-hydroxy-3-ethoxyphenyl) acryloyl) hepta-4,6-dien-1-yl)isoindoline-1,3-dione (K3F23). Yellow powder, yield 30%. Elemental analysis for C_32_H_29_NO_6_ (523.58 g/mol): calc. C 73.41% H, 5.58%, N 2.69%; found: C 73.62%, H 5.62%, N 2.60%. LC-MS (ESI): m/z 524.6 [M + H]^+^. ^1^H NMR (MeOD-*d*_4_) δ keto-enol form: 7.19 (H-3, d (^3^J_HH_ 15.3), 2H), 7.70 (H-4, d (^3^J_HH_ 15.3), 2H), 7.21 (H-6, d (^3^J_HH_ 8 Hz), 2H), 7.00 (H-8, dd, (^3^J_HH_ 8 Hz; ^4^J_HH_ 2 Hz) 2H), 7.20 (H-9, m, 2H), 7.31 (H-10, m, 2H), 2.71 (H-11, t (^3^J_HH_ 7 Hz), 2H), 1.98 (H-12, m, 2H), 3.86 (H-13, t (broad), 2H), 7.89 (H-16, m, 2H), 7.82 (H-17, m, 2H). ^13^C NMR (MeOD-*d*_4_) δ 110.6 (C-1), 183.2 (C-2), 119.4 (C-3), 142.0 (C-4), 134.2 (C-5), 112.6 (C-6), 160.1 (C-7), 116.7 (C-8), 129.8 (C-9), 120.2 (C-10) 23.7 (C-11), 31.6 (C-12), 38.3 (C-13), 168.6 (C-14), 134.8 (C-15), 123.4 (C-16), 134.7 (C-17).

(2-((4Z,6E)-5-hydroxy-7-(4-acetyl-3-methoxyphenyl)-4-((E)-3-(4-hydroxy-3-ethoxyphenyl) acryloyl) hepta-4,6-dien-1-yl)isoindoline-1,3-dione (K3F24). Light orange powder, 45% yield. Elemental analysis for C_36_H_33_NO_10_: calc. C 67.60%, H 5.20%, N 2.19%; found C 67.25%, H 5.35%, N 2.15%. LC-MS-IT m/z 640.8 (M + H)+. ^1^H NMR (CDCl_3_): 7.02 (H-3, d (^3^J_HH_ 15.6 Hz), 2H), 7.76 (H-4, d (^3^J_HH_ 15.6 Hz), 2H), 7.24 (H-6, d (^3^J_HH_ 7.8 Hz), 2H), 7.10 (H-9, d (^3^J_HH_ 7.8 Hz), 2H), 7.21 (H-10, dd (^3^J_HH_ 7.8 Hz, ^4^J_HH_ 3 Hz), 2H), 2.65 (H-11, t (^3^J_HH_ 7 Hz), 2H), 2.05 (H-12, m, 2H), 3.89 (H-13, t (broad), 2H), 7.86 (H-16, m, 2H), 7.80 (H-17, m, 2H). ^13^C NMR (CDCl_3_) δ keto-enol form: 110.5 (C-1), 183.4 (C-2), 120.4 (C-3), 141.6 (C-4), 134.2 (C-5), 111.9 (C-6), 151.4 (C-7), 141.9 (C-8), 121.2 (C-9), 123.2 (C-10) 23.9 (C-11), 31.5 (C-12), 38.2 (C-13), 168.7 (C-14), 134.8 (C-15), 123.4 (C-16), 134.7 (C-17).

(2-((4Z,6E)-5-hydroxy-7-(4-hydroxyphenyl)-4-((E)-3-(4-hydroxy-3-ethoxyphenyl) acryloyl) hepta-4,6-dien-1-yl)isoindoline-1,3-dione (K3F31) Orange powder, 40% yield. Elemental analysis for C_30_H_25_NO_6_: calc. C 72.72%, H 5.09%, N 2.83%) C 73.60%, H 5.21%, N 2.75%. LC-MS-IT m/z 496.6 (M + H)^+^. ^1^H NMR (DMSO-*d*_6_) δ 7.00 (H-3, d (^3^J_HH_ 15.9 Hz), 2H), 7.58 (H-4, d (^3^J_HH_ 15.9 Hz), 2H), 7.54 (H-6/H-10, d (^3^J_HH_ 8 Hz), 4H), 6.79 (H-7/H-9, d(^3^J_HH_ 8 Hz), 4H), 2.65 (H-11, t (^3^J_HH_ 7 Hz), 2H), 1.83 (H-12, m, 2H), 3.77 (H-13, t (broad), 2H), 7.88 (H-16, m, 2H), 7.85 (H-17, m, 2H). ^13^C NMR (DMSO-*d*_6_) δ keto-enol form: 110.8 (C-1), 183.3 (C-2), 117.4 (C-3), 142.1 (C-4), 126.7 (C-5), 131.1 (C-6/C-10), 116.3 (C-7/C-9), 160.4 (C-8), 22.8 (C-11), 26.1 (C-12), 37.6 (C-13), 168.7 (C-14), 134.8 (C-15), 123.4 (C-16), 134.7 (C-17).

### 4.3. Acid-Base Behavior and Stability in Physiological Conditions

UV-vis spectrophotometric measurements were performed using Jasco V-570 spectrophotometer at 25.0 ± 0.1 °C in the 200–600 nm spectral range employing 1 cm quartz cells. Owing to the poor water solubility of the compounds, a methanol mother solution (5 × 10^−3^ M) was diluted in water in order to give 50 µM solutions used for pH-metric titrations. The pH value was varied by adding small amounts of concentrated NaOH or HCl, 25 points in the pH range 3–10. A constant ionic strength of 0.1 M (NaNO_3_) was maintained in all the experiments. Each titration was performed at least three times. The overall protonation constants (logβ_LH_) were evaluated from spectrophotometric data using the software HypSpec [48].

Kinetic stability studies were performed at 37 °C in darkness, the change in absorbance in the 200−600 nm range over an overall period of 8 h was estimated for all the K3F samples by UV-vis spectroscopy. 100 μM solutions of the ligands were prepared in 0.1 M Tris-HCl buffer (pH 7.4) with constant ionic strength (0.1 M NaNO_3_). Spectra were recorded every 30 min. All profiles were linearized by hyperbolic function (Equation (1)), which represents an empirical model that well describes drug decomposition or release [49].

t/f_%_ = at + b (1)

where f_%_ is the fraction of residual compound at time t (min) expressed as percentage referred to starting concentration at time zero.

### 4.4. Antioxidant Activity (DPPH Assay)

The antioxidant activity of the compounds was evaluated in terms of hydrogen donating or radical scavenging ability, using the stable DPPH radical (1,1-diphenyl-2-picrylhydrazyl radical). A variable amount (15, 30, 45, 75, 105, and 150 µL) of a methanol solution (1.2 mM) of each compound was placed in a cuvette, and 3 mL of a 60 µM methanol solution of DPPH was added. Absorbance measurements were initiated immediately. The decrease in absorbance at 517 nm was monitored every minute up to 5′, then every 5′ up to 30′ and every 30′ until reaction reaches completion and absorbance stabilizes attaining a plateau after 120′. Methanol was used to zero the spectrophotometer. The absorbance of the DPPH radical without K3F compound, i.e., the control, was measured daily, and concentration was calculated applying Equation (2) [50]

(2) $$\left[{\mathrm{DPPH}}^{\cdot }\right]=\frac{A-1.006}{10970}$$

The percentage of inhibition (%In) of the DPPH radical by each sample was calculated according to the formula

(3) $$\%\mathrm{In}=\frac{A_{0}-A_{t}}{A_{0}}\times 100$$

where A_0_ represents the absorbance of the control (DPPH radical) at time 0, while A_t_ refers to the absorbance of the mixture DPPH/antioxidant at time t (120 min). Values of absorbance were corrected taking into account volume dilution and all determinations were performed in triplicate.

### 4.5. Cell Lines and Treatments

Human prostate cancer PC3 cells (CRL 1435, ATCC, Manassas, VA, USA) were grown in Ham’s F12 nutrient medium, human prostate cancer DU145 cells (HTB-81, ATCC, Manassas, VA, USA) in RPMI 1640 medium and human colorectal carcinoma HCT116 cells (CCL-247, ATCC, Manassas, VA, USA) in IMDM medium. All media (Biowest, Nuaillé, France) were supplemented with 10% Foetal Bovine Serum (Gibco-Thermo Fisher Scientific, Waltham, MA, USA), L-glutamine, penicillin, and streptomycin (Euroclone, Milan, Italy), and grown at 37 °C in a humidified 5% CO_2_ atmosphere.

Synthesized curcumin, K3F compounds and docetaxel (MedChem Express, Monmouth Junction, NJ, USA) were dissolved in DMSO (Sigma-Aldrich, St. Louis, MO, USA) and added to cell medium at the concentrations described in the text for 48 h. DMSO was used as control. Docetaxel IC50 values were calculated through MTT assay and estimated as 17 nM and 30 nM in DU145 and PC3, respectively.

### 4.6. Cell Viability Assay

The inhibition of cell proliferation was measured by colorimetric MTT assay [51] and the concentration at which cellular growth is inhibited by 50% (GI50) was determined. Briefly, cells were seeded into 96-well plates at a density of 2000 cells/well. The following day, cells were treated with the indicated compounds and analyzed for cell viability after 48 h. 5 mg/mL thiazolyl blue tetrazolium bromide (Sigma-Aldrich, St. Louis, MO, USA) was added to each well at a final concentration of 0.5 mg/mL and the plates were incubated at 37 °C for 2 h. Medium containing unconverted MTT was removed and 100 µL MTT solvent (4 mM HCl, 0.1% NP-40 in isopropyl alcohol) was added to each well. The plate was gently rotated on an orbital shaker in the dark for 15 min, before the measurements of absorbance at 570 nm. The viability of untreated cells was arbitrarily set at 100%.

The ability of K3F21 and docetaxel to act synergistically with regard to growth inhibition was determined using CompuSyn software (ComboSyn, Inc., freely available at http://www.combosyn.com) [34]. Combination index (CI) and drug reduction index (DRI) were calculated for each concentration of drugs mixture, as indicated in the text. CI <1, =1 or >1 indicates synergism, additive effect, or antagonism, respectively. DRI >1 or <1 indicates favorable or not favorable dose-reduction.

### 4.7. Anchorage-Dependent and -Independent Colony Formation Assay

For anchorage-dependent colony formation assay, 1000 PC3 or 500 DU145 cells were plated in 6-well plates. Following overnight incubation, the cells were treated with curcumin or K3F21 (2, 5, 10, 20 µM) or with DMSO, as control. After 7–10 days, the plates were washed, fixed and stained with 0.5% crystal violet solution in 20% Methanol. Visible colonies with radius ≥3 pixels were counted with automated colony counter (OpenCFU 3.9.0 software, freely available at http://opencfu.sourceforge.net/) and plotted against curcumin concentrations. Colony numbers were normalized as a percentage of colonies formed following DMSO treatment.

For anchorage-independent colony formation assay, 0.6% low melt agarose gel with 10% FBS in appropriate complete cell growth medium was prepared and added to the wells of a twelve-well culture dish as a base agar. 1200 cells per well were plated in 0.25% (PC3) or 0.2% (DU145) agarose gel in appropriate complete cell growth medium supplemented with varying concentrations of curcumin and K3F21 (5, 10, 20 µM) or with DMSO, as control. PC3 and DU145 cells were allowed to grow at 37 °C for three weeks (PC3) or five weeks (DU145). The effect of the drugs on anchorage-independent growth was determined by colony growth. Colonies were stained with 0.07% crystal violet and formed colonies were imaged. The numbers of colonies with radius ≥ 7 pixels were counted with automated colony counter (OpenCFU 3.9.0) and average number of colonies was plotted against drug concentrations. Colony numbers were normalized as a percentage of colonies formed following DMSO treatment.

### 4.8. Cell Cycle Analysis

Cells were harvested after 48 h drug treatments and DNA distribution analysis of cells stained with propidium iodide solution (propidium iodide 25 µg/mL, Na-Citrate 3.4 mM, NaCl 9.65 mM, NP-40 0.03%) was performed by an Epics cytofluorimeter (Beckman Coulter Srl, Milan, Italy) [52,53].

### 4.9. Cell Migration Assay

4 × 10^5^ prostate cancer cells were plated in a 12-well plate. Approximately 24 h later, when the cells were 100% confluent, the monolayer was scratched using a 200 µL pipette tip. Medium and non-adherent cells were removed and new medium containing 1% FBS and GI50 concentrations of Curcumin, K3F21 and DTX was added. Cells were observed under the microscope and the inhibition of migration was assessed when the wound in the control was closed, after 16 h and 40 h in DU145 and PC3, respectively. Three biological replicates were performed. Three random pictures were taken for each treated well per experiment and the areas of the wounds measured using ImageJ software. The percentage of wound recovery was then calculated using the formula: (final area-initial area)/initial area × 100%.

### 4.10. Protein Extracts and Western Bot

Whole-cell protein extracts were prepared by resuspending cells into 1X SDS sample buffer (25 mM Tris-HCl pH 6.8, 1.5 mM EDTA, 20% glycerol, 2% SDS, 5% β-mercaptoethanol, 0.0025% Bromophenol blue). Equivalent amounts of cellular extracts were resolved by SDS-PAGE, electrotransferred to PVDF membrane (GE Healthcare Italia, Milan, Italy) and immunoblotted with the following primary antibodies: anti-phospo-H2AX (sc-101696), anti-p53 (DO1) (sc-126), anti-actin (I19) (sc-1616) and anti-PARP (sc-8007) antibody from Santa Cruz Biotecnology, Inc, Dallas, TX, USA.; phospho-specific AKT antibody S473 (#9018) and T308 (#9275), Akt antibody (#9272 and #2967), phospho-specific ERK1/2 antibody T202/Y204 (#4370), ERK1/2 antibody (#4669), phospho-specific p38 MAPK antibody T180/Y182 (#9211), and p38 antibody (#9212) were purchased from Cell Signaling Technologies, Danvers, MA, USA. Membranes were blotted and scanned with Bio-Rad ChemiDocTM Touch Imaging System (Bio-Rad, Hercules, CA, USA), using chemiluminescent detection reagents from Cyanagen, Bologna, Italy (Westar ηC and Supernova HRP substrates for Western blotting).

### 4.11. RNA Extraction and RT-qPCR

RNA was extracted from cells using RNeasy kit (Qiagen, Venlo, The Netherlands) and reversed transcribed with a Moloney murine leukemia virus reverse transcriptase (Promega, Madison, WI, USA). Quantitative real-time PCRs were performed using SsoAdvanced Universal SYBR Green Supermix (Bio-Rad, Hercules, CA, USA) on a Bio-Rad CFX Connect™ real-time PCR detection system. Primer sequences are available upon request. The housekeeping gene Rpl21 was used as a loading control. The relative fold change enrichments of real-time PCR samples were calculated with the formula 2^−(ΔΔCt)^, where −(ΔΔCt) = −[(Ct target − Ct Rpl21)treated − (Ct target − Ct Rpl21)DMSO].

### 4.12. Statistical Analysis

Experiments were performed at least in biological triplicates and graphs represent mean ± SEM values. All statistical analyses were performed using GraphPad PRISM v8 software (GraphPad Software, La Jolla, CA, USA), using two-tailed *t*-tests or one-sample *t*-tests, as appropriate. *p* values of *p* < 0.05 were considered to be statistically significant (*), *p* < 0.01 (**), *p* < 0.001 (***), and *p* < 0.0001 (****).

## Supplementary Materials

Supplementary materials can be found at http://www.mdpi.com/1422-0067/20/1/28/s1. Table S1: Logarithms of Protonation Constant (logβLH) and pK_a_ values, Figure S1: UV-vis spectra of K3F21 on pH variation, Figure S2: Species distribution curves for ligands in the 3–9 pH range at [L]tot = 2 × 10^−4^ M, Figure S3: Biological activity of K3-derivatives on human colorectal HCT116 cancer cells, Figure S4: Cell cycle analysis of PC3 and DU145 cells, Figure S5: Protein expression analysis of PCa cells following DTX administration. Figure S6: AIG colony assay in PCa cells.

## Abbreviations

PCa	Prostate cancer
AR	Androgen receptor
DTX	Docetaxel
DMSO	Dimethylsulfoxide
CRPC	Castration-resistant PCa
AIG	Anchorage independent growth
