# Supplementary material for: Potent Anti-Cancer Properties of Phthalimide-Based Curcumin Derivatives on Prostate Tumor Cells
Source: Int J Mol Sci. 2018 Dec 21;20(1):28. doi: 10.3390/ijms20010028 (PMC6337497; doi:10.3390/ijms20010028)
Supplement: Supplementary file 1 [file ijms-20-00028-s001.pdf]

	K3F21	K3F23	K3F24	K3F31	K3F33
$Log\beta_{11}$	9.84 (2)	8.49 (3)	8.52 (3)	10.88(2)	7.97 (3)
$Log\beta_{12}$	17.80 (2)	/	/	20.29(2)	/
$Log\beta_{13}$	24.6 (1)	/	/	26.84(4)	/
pK_{a1}	6.8 (1)	8.49 (3)	8.52 (4)	6.55(4)	7.97 (3)
pK_{a2}	7.96 (1)	/	/	9.41(2)	/
pK_{a3}	9.84 (2)	/	/	10.88(2)	/

$log\beta_{LH}$ values refer to the following equilibria for K3F21 and K3F31:

$$Log\beta_{11} \quad L^{3-} + H^+ \rightleftarrows HL^{2-}$$
$$Log\beta_{12} \quad L^{3-} + 2H^+ \rightleftarrows H_2L^-$$
$$Log\beta_{13} \quad L^{3-} + 3H^+ \rightleftarrows H_3L$$

$log\beta_{LH}$ value refers to the following equilibrium for K3F23, K3F24, and K3F33:

$$Log\beta_{11} \quad L^- + H^+ \rightleftarrows HL$$

Supplementary Table 1. Logarithms of Protonation Constant ($log\beta_{LH}$) and pK_a values. $Log\beta_{LH}$ and pK_a values were calculated with HypSpect [15] from spectrophotometric titrations performed at 25°C, I = 0.1 M ($NaNO_3$). For K3F21 and K3F31, pK_a values were calculated from $log\beta$ values as follows: $pK_{a3} = log\beta_{11}$; $pK_{a2} = (log\beta_{12} - log\beta_{11})$; $pK_{a1} = (log\beta_{13} - log\beta_{12})$.

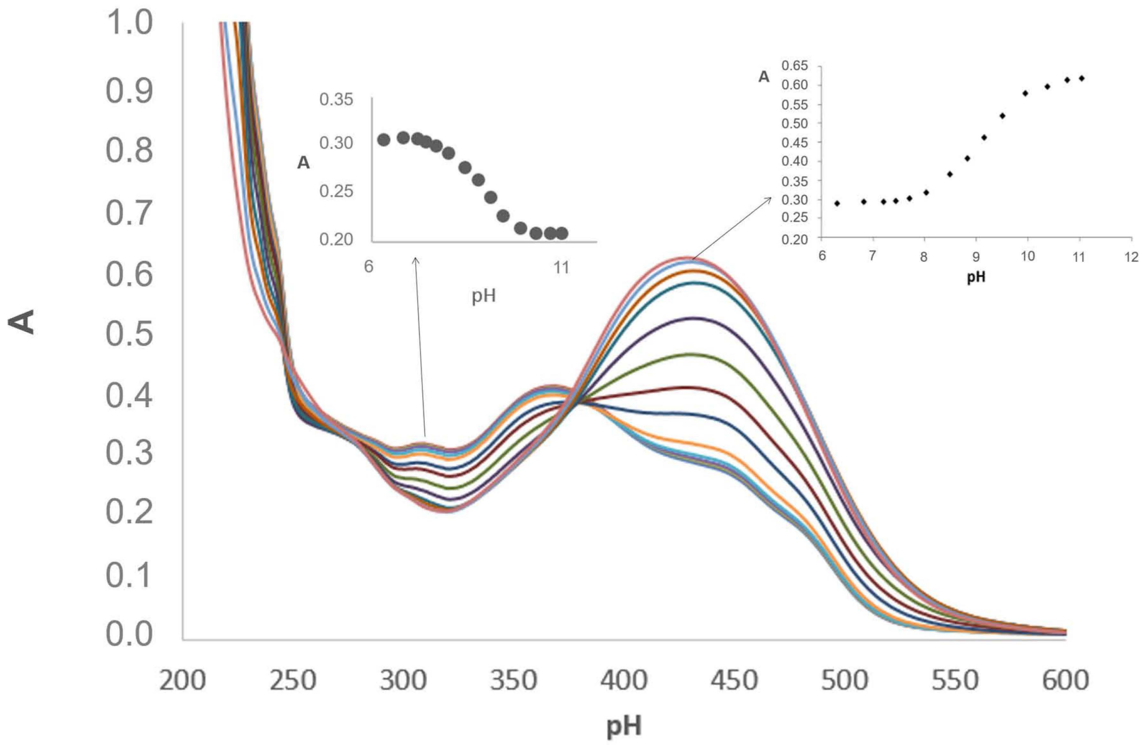

Supplementary Figure 1. UV-vis spectra of K3F21 on pH variation. The inserts represent the plot of absorbance (A) vs. pH at λ 310 nm and 420 nm respectively ([K3F21]aq = 50 μM; 25 °C).

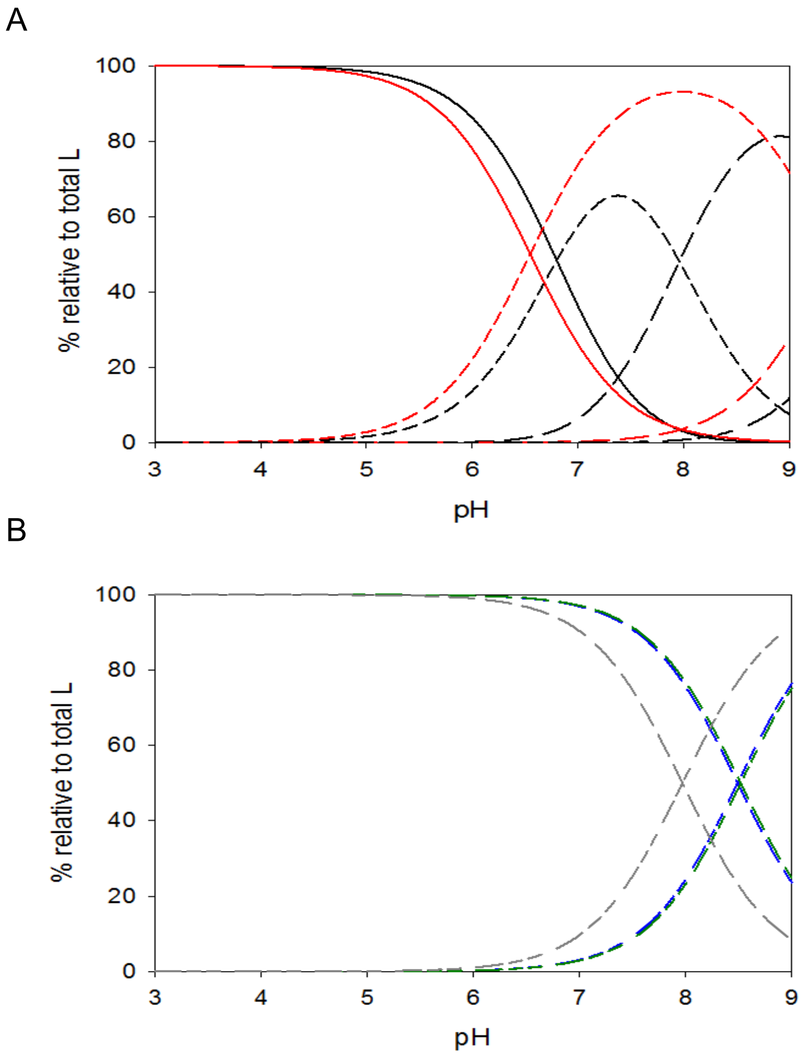

Supplementary Figure 2. Species distribution curves for ligands in the 3-9 pH range at [L]tot = 2×10-4 M. Solid line (____) H3L, short dashed line (- - -) H2L, long dashed line (___ ___) HL, long-short-short dashed line (___ - -) L (charges are omitted for clarity). Panel A: black K3F21 and red K3F31. Panel B: blue K3F23, green K3F24, and dark grey K3F33.

A

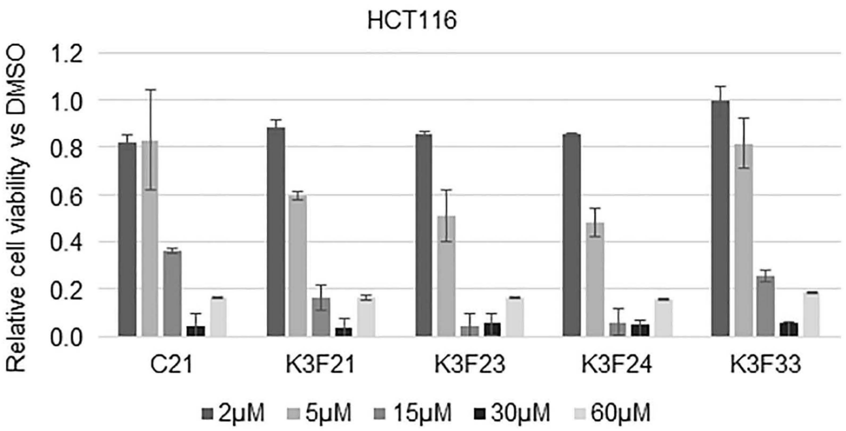

B

	GI50 (μM)	SEM
C21	12.81	0.33
K3F21	4.90	0.67
K3F23	7.01	0.62
K3F24	6.73	0.12
K3F33	9.36	0.97

C

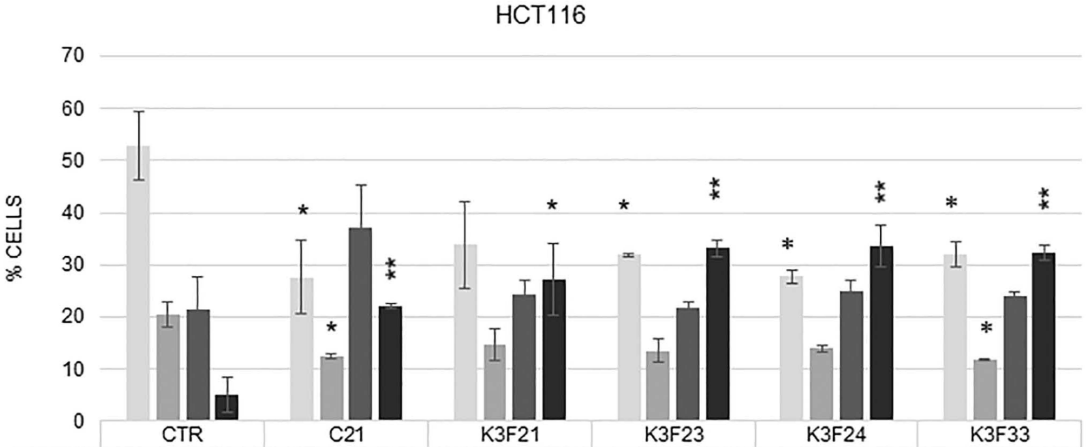

	CTR	C21	K3F21	K3F23	K3F24	K3F33
G0/G1	52.85	27.65	33.85	31.85	27.70	31.95
S	20.45	12.55	14.75	13.45	13.90	11.75
G2/M	21.50	37.00	24.25	21.80	25.00	24.05
SubG1	5.10	21.95	27.20	33.15	33.70	32.30

G0/G1 S G2/M SubG1

Supplementary Figure 3. Biological activity of K3-derivatives on human colorectal HCT116 cancer cells. (**A**) Dose-dependent effect (GI50) of K3-derivatives administration for 48 hours on cell growth of HCT116 colorectal cancer cells. The bars represent the media of five independent experiments -/+ SEM. (**B**) GI50 values, SEM and statistical significance compared to curcumin have been indicated for HCT116 cells. (**C**) Distribution between cell cycle phases following administration for 48 hours of DMSO (CTR) or the indicated K3-derivatives in HCT116 cells. The bars represent the media of three independent experiments -/+ SEM. P values refer to the comparison between K3-derivatives and CTR samples. P value * < 0.05, ** < 0.01, *** < 0.001, **** < 0.0001.

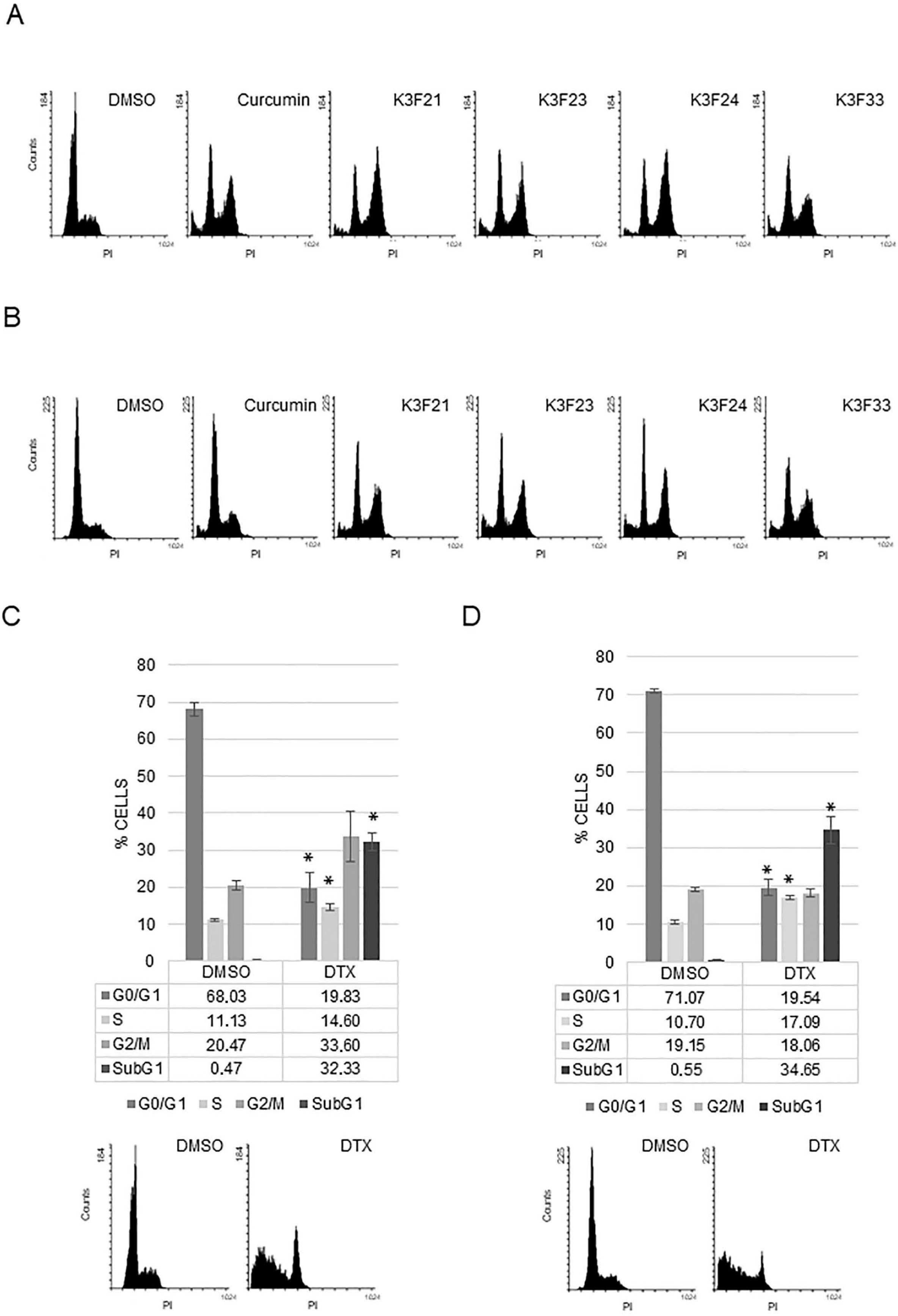

Supplementary Figure 4. Cell cycle analysis of PC3 and DU145 cells. (**A** and **B**) Representative images of cytofluorimetric cell cycle analysis of PC3 and DU145 cells treated for 48h with GI50 doses of the indicated molecules. (**C** and **D**) Cell distribution between cell cycle phases following administration for 48 hours of DMSO and DTX on PC3 and DU145 cells at GI50 concentration. The bars represent the mean of three independent experiments ± SEM. Representative images of cell cycle analysis are shown in lower panels. P values refer to the comparison between DTX and DMSO samples: * < 0.05, ** < 0.01, *** < 0.001, **** < 0.0001.

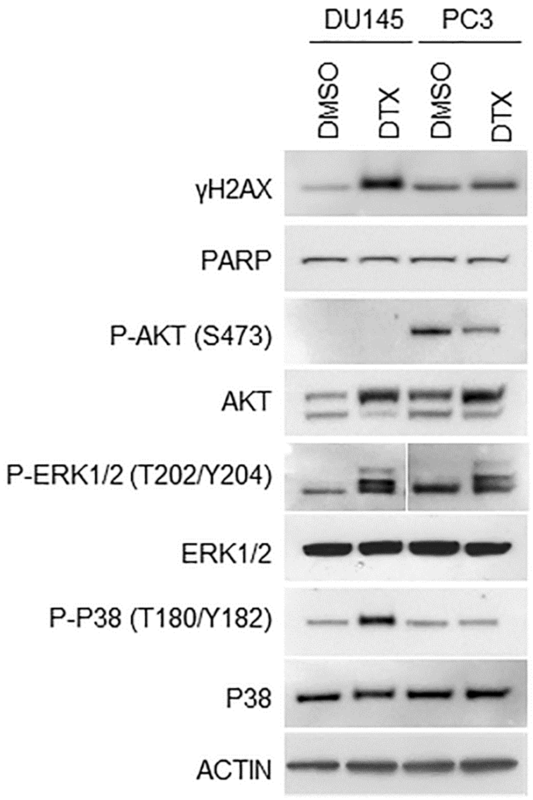

Supplementary Figure 5. Protein expression analysis of PCa cells following DTX administration. Total cellular lysates from DU145 and PC3 cells treated with DMSO and DTX for 48 hours at GI50 dose were analysed by western blot with the indicated antibodies.

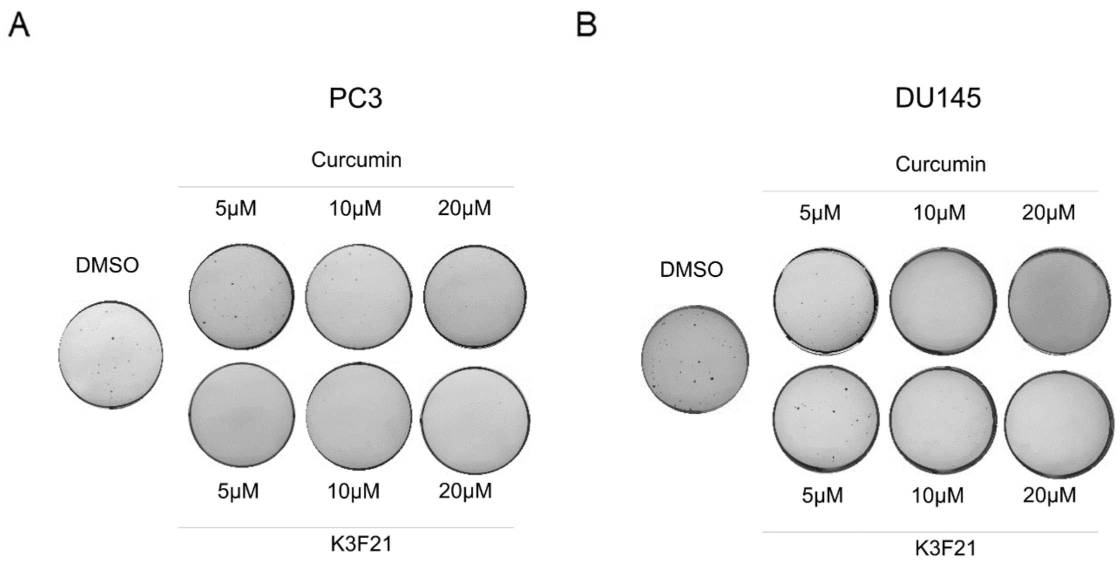

Supplementary Figure 6. AIG colony assay in PCa cells. Representative images of anchorage-independent colony assays performed in the presence of increasing doses of curcumin and K3F21 in PC3 (**A**) and DU145 (**B**) cells.
